# VEGF released by deferoxamine preconditioned mesenchymal stem cells seeded on collagen-GAG substrates enhances neovascularization

**DOI:** 10.1038/srep36879

**Published:** 2016-11-10

**Authors:** Elizabeth A. Wahl, Thilo L. Schenck, Hans-Günther Machens, Elizabeth R. Balmayor

**Affiliations:** 1 Department of Plastic Surgery and Hand Surgery, University Hospital rechts der Isar, Technische Universität München, Munich, 81675, Germany; 2 Institute for Advanced Study, Technische Universität München, Garching, 85748, Germany

## Abstract

Hypoxia preconditioning of mesenchymal stem cells (MSCs) has been shown to promote wound healing through HIF-1α stabilization. Preconditioned MSCs can be applied to three-dimensional biomaterials to further enhance the regenerative properties. While environmentally induced hypoxia has proven difficult in clinical settings, this study compares the wound healing capabilities of adipose derived (Ad) MSCs seeded on a collagen-glycosaminoglycan (GAG) dermal substrate exposed to either environmental hypoxia or FDA approved deferoxamine mesylate (DFO) to stabilize HIF-1α for wound healing. The release of hypoxia related reparative factors by the cells on the collagen-GAG substrate was evaluated to detect if DFO produces results comparable to environmentally induced hypoxia to facilitate optimal clinical settings. VEGF release increased in samples exposed to DFO. While the SDF-1α release was lower in cells exposed to environmental hypoxia in comparison to cells cultured in DFO *in vitro*. The AdMSC seeded biomaterial was further evaluated in a murine model. The implants where harvested after 1 days for histological, inflammatory, and protein analysis. The application of DFO to the cells could mimic and enhance the wound healing capabilities of environmentally induced hypoxia through VEGF expression and promises a more viable option in clinical settings that is not merely restricted to the laboratory.

Treatment of non-healing chronic wounds, such as venous leg ulcers, diabetic foot ulcers, and pressure ulcers, has a major impact on the healthcare system in the US with about 2% of the population needing treatment and costs exceeding $50 billion per year^1^. With an aging population and a likelihood that the majority of the costs come from patients over 65, the costs could further increase^2^. In addition, these wounds have a negative effect on the quality of life of the patients^3^,^4^, for example, diabetic related amputations^5^,^6^ and mortality rates reaching 68% from advanced pressure ulcers^7^.

Mesenchymal stem cells (MSCs) are known for their regenerative potential and have been studied for implementation in clinical settings. However, the MSC niche consists of varying low oxygen conditions. When they are cultured *in vitro* with the intention of being used for regenerative purposes they are often subjected to atmospheric oxygen tensions. MSCs maintained at low oxygen conditions have shown improved growth, cytokine release, and stability, as well as the regulation of hypoxia inducible factor (HIF)-1 in order to improve their performance in therapy^8^,^9^,^10^.

MSCs are currently being studied in order to treat chronic wounds, although a high number of cells are often needed as they can migrate after implantation^11^. Utilizing a biomaterial can impede this migration and increase the concentration of secreted factors from the cells to the local area. In chronic wounds, one of the main inhibiting factors is the lack of vasculature due to the inability of the vessels to regrow fast enough to stabilize the wound. Cell signaling can help initiate angiogenesis while a biomaterial can create a platform for revascularization^12^.

Scaffolds for dermal regeneration have been widely used for the treatment of skin wounds; however, their clinical use is limited to cases where autologous tissue transplantation is not possible (e.g., massive burns). In order to improve the efficacy of dermal scaffolds, the application of MSCs on the material has been suggested^13^. MSCs have shown vast regenerative capabilities in different tissue defects including non-healing wounds^14^. Thus, the combined use of MSC-seeded scaffolds is a matter of intense research in the field of tissue engineering and regeneration. Furthermore, the optimization of such hybrid implants is particularly relevant for translational approaches.

In this context, hypoxic preconditioning has been shown to enhance the regenerative potential of MSCs after *in vivo* implantation. Adipose derived (Ad)MSCs can stimulate new vessel growth in low oxygen conditions through the increased expression of the transcription factor, hypoxia inducible factor (HIF)-1α, which also upregulates vascular endothelial growth factor (VEGF)^16^ and stromal cell-derived factor (SDF)-1α^17^. VEGF provides signaling crucial to angiogenesis, promoting revascularization in wound healing, while SDF-1α contributes to the migration and recruitment of endothelial progenitor cells to the wound area further promoting neovascularization.

Iron chelating deferoxamine mesylate (DFO) stabilizes HIF-1α under normoxic conditions making it a suitable substitute for environmental hypoxia. In diabetics, the expression of VEGF under hypoxic conditions is impaired; therefore, DFO has successfully been used to treat wounds^18^. As AdMSCs exposed to low oxygen tensions have shown higher rates of neovascularization and, thus, improved wound healing, we evaluated the impact of a chemically induced stabilization of HIF-1α through DFO in comparison to environmentally induced hypoxia. This could make the treatment easier to translate into a clinical setting. We found that cells preconditioned with DFO exhibited an increase in VEGF release *in vitro* and improved neo-vascularization in a murine wound healing model. The cells were housed in a collagen-glycosaminoglycan (GAG) crosslinked dermal scaffold in order to facilitate new vessel growth and promote an increase in cellular secretions.

## Results

**Figure 1:**
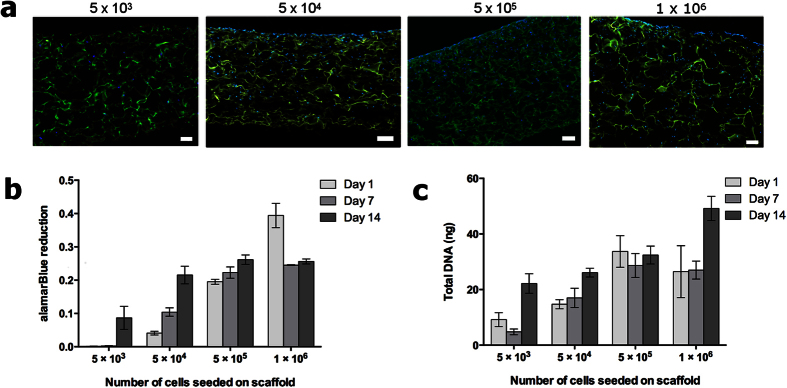
Optimization of the cellular density. The nuclei of the cells in the scaffolds (green autofluorescence) were stained with DAPI (blue) 14 days after seeding (**a**). Cross sections (5 μm) of 10 mm scaffolds were imaged with the top of the scaffold being the seeding side. The metabolic activity (alamarBlue) (**b**) and the amount of dsDNA (PicoGreen) (**c**) of the same sample were measured after 1, 7, and 14 days. Although the scaffolds seeded with 1 × 10^6^ cells showed growth, the DNA content decreased, which may have been due to overcrowding and low nutrient supply. Therefore, as scaffolds seeded with 5 × 10^5^ showed the most stability over the three time points this was the chosen seeding density for all further experiments. All experiments were performed in triplicate with samples from three donors. Scale bars represent 100 μm.

AdMSCs were seeded on 10 mm collagen-GAG scaffolds at different densities to determine the optimal seeding density. This was estimated via their metabolic activity in regards to the cellular proliferation over time (Fig. 1). At all seeding concentrations, cells were distributed throughout the scaffold (Fig. 1a). The metabolic activity (Fig. 1b) and total dsDNA (Fig. 1c) was quantified over a period of two weeks. During this time 5 × 10^5^ cells were determined to be the optimal seeding density for the size of the scaffold as the cells maintained a stable rate of metabolic activity over two weeks. This corresponds to a density of 6.4 × 10^5^ cells per cm^2^. With a seeding density of 5 × 10^3^ cells, the metabolic activity was very minimal until day 14. Scaffolds with 5 × 10^4^ cells exhibited good cellular growth but after one day the metabolic activity was lower than desired. At a density of 1 × 10^6^ cells, the metabolic activity decreased between days 1 and 7, although the total dsDNA increased at day 14, indicating the cells may have been overcrowded.

**Figure 2:**
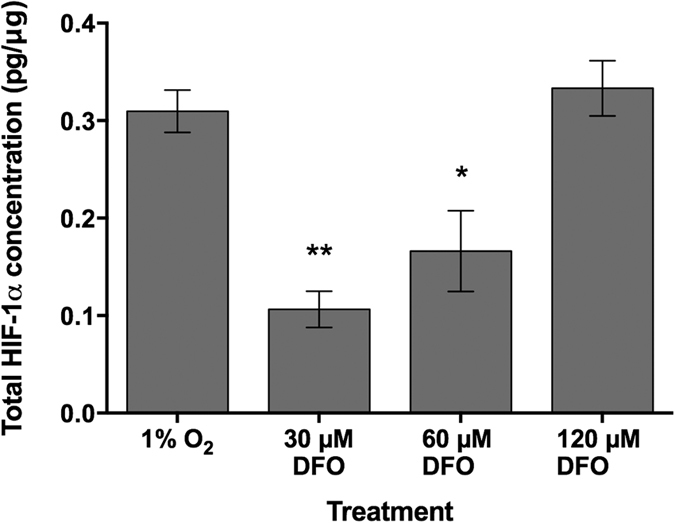
Total HIF-1α protein quantification from cell lysates *in vitro.* The HIF-1α concentration was quantified from cell lysates by ELISA and a BCA total protein quantification assay after 12 hours in culture. No HIF-1α activity was detected in the normoxic control groups and, therefore, is not presented in the graph. All DFO treated groups were incubated under standard cell culture conditions. Significance is indicated by *p ≤ 0.05, **p ≤ 0.01 when compared to the 1% O_2_ treatment.

Cells seeded on collagen-GAG scaffolds were exposed to DFO and environmental hypoxia. The total HIF-1α from cell lysates was quantified after 12 and 24 hours in order to determine if the DFO could stabilize HIF-1α and if there was a difference in release due to the concentration of DFO or environmental hypoxia. AdMSC seeded scaffolds that were exposed to DFO were incubated under standard cell culture conditions as well as seeded cells without DFO that were utilized as a control. No HIF-1α expression could be detected (data not shown) from the control scaffolds. Furthermore, there was no HIF-1α expression exhibited from samples after 24 hours in culture, as expected. No significant differences could be seen between AdMSC seeded collagen-GAG that was exposed to 1% O_2_ and 120 μM DFO (Fig. 2). Cells exposed to 30 or 60 μM of DFO showed lower expression of HIF-1α indicating that the effect is concentration dependent and 120 μM is sufficient to mimic that of cells under hypoxic conditions.

**Figure 3:**
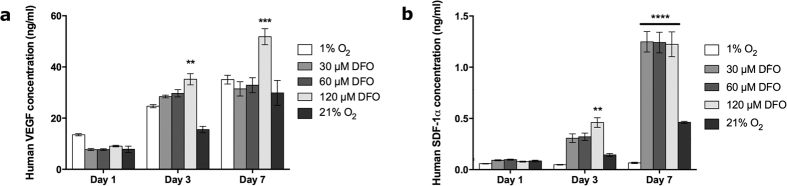
VEGF and SDF-1α release from scaffolds *in vitro.* VEGF and SDF-1α concentrations were quantified from cell culture supernatants by ELISA. The release of VEGF from AdMSCs exposed to 120 μM DFO was higher after three and seven days (**a**). Alternatively, higher amounts of SDF-1α were released from cells when exposed to DFO, although the concentration did not affect the release in a significant way (**b**). All DFO treated groups were incubated under standard cell culture conditions. Significance is indicated by **p ≤ 0.01, ***p ≤ 0.001, ****p ≤ 0.0001 when compared to the 21% O_2_ control.

To determine the potential for HIF-1α related paracrine factors, the release of VEGF and SDF-1α from the AdMSCs was determined by ELISA protein quantification. No significant differences could be seen after one day between treatment groups from either protein. After days three and seven, a significantly higher amount of VEGF was secreted from cells exposed to 120 μM DFO than those under standard cell culture conditions (Fig. 3a). There was a significantly lower amount of SDF-1α released from cells under environmental hypoxia compared to all other conditions after seven days. DFO alone showed a strong release of SDF-1α after seven days, which did not appear to be concentration dependent. After three days, a significant increase of release from cells exposed to 120 μM DFO in comparison to 21% O_2_ was detected (Fig. 3b).

With the *in vitro* results from the different concentrations of DFO, 120 μM was used for the *in vivo* mouse wound healing model. This was the lowest concentration tested to mimic the expression and release of angiogenic factors when compared to hypoxic treatment. A dermal wound was inflicted and a collagen-GAG scaffold with either preconditioned cells (120 μM DFO and 1% O_2_), control cells (21% O_2_), or empty scaffolds (without cells and scaffolds incubated with 120 μM DFO) was sutured into place.

**Figure 4:**
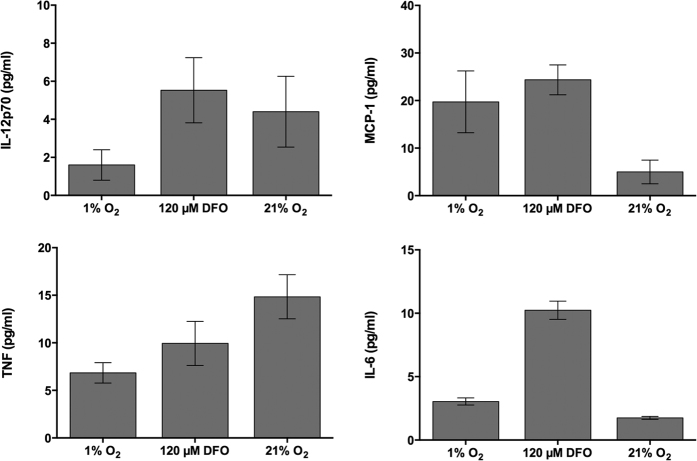
Inflammatory response *in vivo.* A flow cytometer analysis was run using serum obtained from the mouse heart 14 days after surgery. Expression of IL-12p70 and TNF did not show any significant differences between treatments. MCP-1 and IL-6 showed higher levels of occurrence in samples that were exposed to DFO. In addition, IL-10 and IFN-γ were analyzed but no expression was seen. All DFO treated groups were incubated in normoxic conditions. Significance is indicated by *p ≤ 0.05, ****p ≤ 0.0001 when compared to the 21% O_2_ control. Error bars indicate the mean ± SD.

No signs of inflammation were detected from the wounds of the mice when the scaffolds were harvested 14 days after implantation. Therefore, in order to determine if there was a systemic inflammatory response from the treatment, blood was collected from the heart and the serum was analyzed by FACS for six inflammatory markers, including Interleukin-10 (IL-10), Interferon-γ (IFN-γ), Interleukin-12p70 (IL-12p70), Tumor Necrosis Factor (TNF), Monocyte Chemoattractant Protein-1 (MCP-1), and Interleukin-6 (IL-6). IL-10 and IFN-γ did not show expression in any of the treatments. IL-12p70 and TNF did not show any significant differences between treatment groups (Fig. 4). MCP-1 showed a higher concentration in mice exposed to DFO treated cells and IL-6 further exhibited a highly significant increase of expression in DFO exposed cells in comparison to those treated in environmental hypoxic or normoxic conditions (Fig. 4).

**Figure 5:**
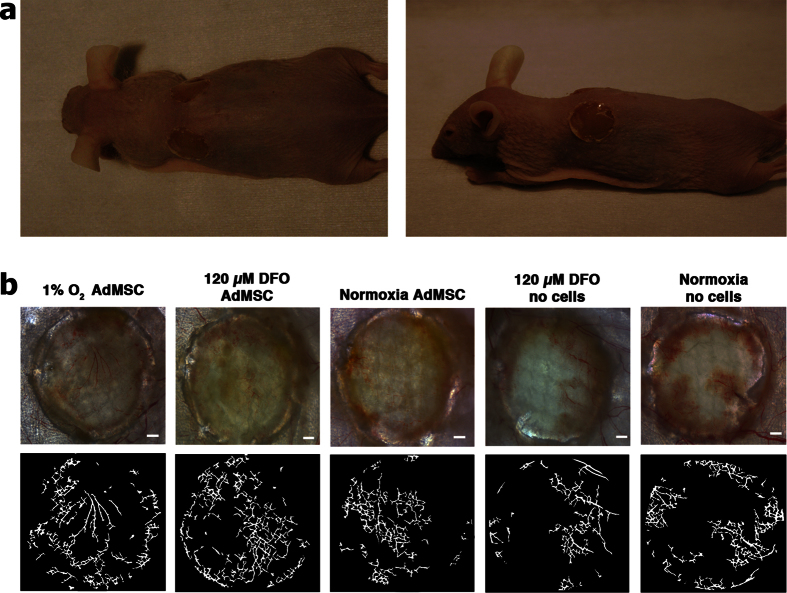
Vascularization of collagen-GAG scaffolds. Nude immunocompetent male mice were wounded with a biopsy punch and a 10 mm collagen-GAG scaffold seeded with cells from treatment and control groups were sutured in place (**a**). The scaffolds were harvested 14 days after implantation and the neovascularization on the underside of the scaffolds was imaged by transillumination. The mesh seen in the image is from the titanium implant to avoid wound contracture (**b** top row). Digital segmentation was performed using VesSeg software to better visualize the vessels in the collagen-GAG scaffold (**b** bottom row). Scale bars indicate 1 mm.

Each animal was photographed after they were euthanized 14 days after surgery with the bandages and sutures removed (Fig. 5a). Scaffolds were then harvested from the mice and the underside was imaged with a transilluminator to view any new vessel growth resulting from the treatment. A digital segmentation of the images, to view the new growth more clearly, revealed a higher density of vessel structures in scaffolds that had AdMSCs exposed to hypoxic conditions and DFO (Fig. 5b). Vessel growth can also be seen when there were no cells and with AdMSCs exposed to normoxic conditions but there are visually less.

**Figure 6:**
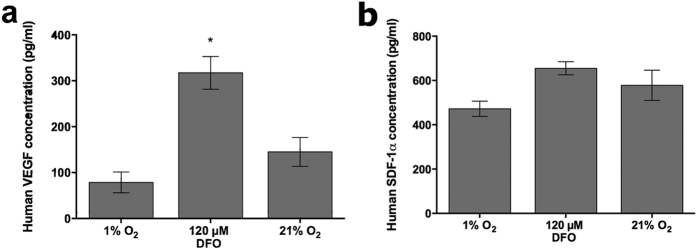
VEGF and SDF-1α release *in vivo.* Collagen-GAG scaffolds harvested from mice after 14 days were evaluated for human VEGF (**a**) and SDF-1α (**b**) concentrations released from the AdMSCs, which were quantified from cell lysates by ELISA. Significantly high levels of VEGF expression were detected from samples exposed to DFO. Significance is indicated by *p ≤ 0.005 when compared with the 21% O_2_ control.

In order to determine if the AdMSCs remained on the scaffolds and were viable, the harvested scaffolds were checked for the presence of human VEGF and SDF-1α. As with *in vitro* results, there was a higher instance of VEGF detected with cells exposed to 120 μM DFO than normoxic or environmentally hypoxic conditions (Fig. 6a). In contrast to *in vitro* results, the levels of SDF-1α remained similar between AdMSC treated groups (Fig. 6b). Scaffolds that did not have cells seeded on them did not show any release of VEGF or SDF-1α, further indicating that antibodies were specific for human proteins (data not shown).

**Figure 7:**
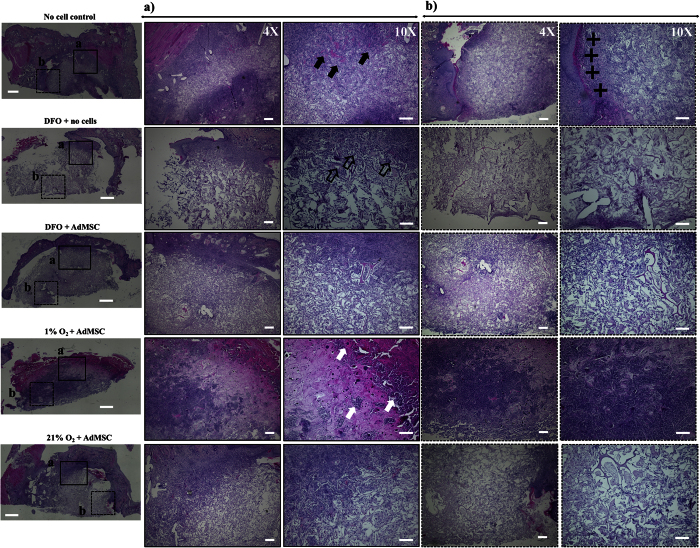
H&E staining of collagen scaffolds 14 days after implantation into the subcutaneous tissue of mice. Representative images selected from N = 4 sections analyzed per group. On the left, an overview of the entire histological section is shown. Two quadrants, (**a,b**) representing the region of interest (ROI) are indicated (scale bar = 1 mm). For better visualization, the ROIs (**a,b**) were photographed at 4x (scale bar = 250 μm) and 10x (scale bar = 150 μm) magnifications and are depicted on the right. In the images corresponding to the ROI (**a**) in the control group (i.e. no cells), black arrows indicate the presence of foreign body giant cells next to mononuclear cell infiltrates. In the image corresponding to the same group but ROI (**b**), marked with (+) is a capsule formation that can be seen around the implantation site. In the group DFO + no cells, ROI (**a**), open arrows indicate the vessel structures that can be seen in the scaffold-to-tissue transition area. In the group 1% O_2_ + AdMSC, ROI (**a**), white arrows indicate multinucleated foreign body giant cells. A dense infiltrate of leukocytes was observed in both ROI (**a**,**b**) for this group.

**Figure 8:**
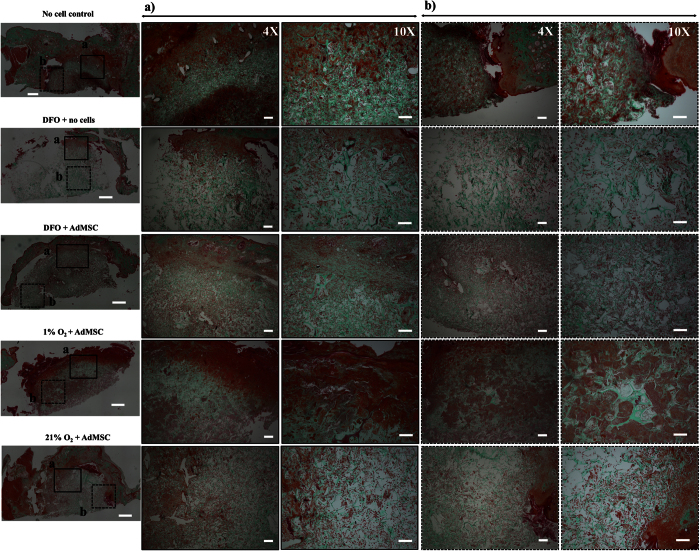
Masson’s Trichrome staining of collagen scaffolds 14 days after implantation into the subcutaneous tissue of mice. Representative images selected from N = 4 sections analyzed per group. On the left, an overview of the entire histology section is shown. Two quadrants have been indicated with (**a,b**) that represent the region of interest (ROI; scale bar = 1 mm). For better visualization, the ROI (**a,b**) were photographed at 4x (scale bar = 250 μm) and 10x (scale bar = 150 μm) magnifications and are depicted on the right.

Histological analysis was performed with all explants collected 14 days post-implantation. Representative images for H&E and Masson’s Trichrome staining are shown in Figs 7 and 8, respectively. The presence of local inflammatory cells can be observed around the scaffold material in the groups where no cells were pre-seeded on the implanted scaffolds (i.e., no cell control and DFO + no cells). This is particularly observable for the control group (i.e., no cells), were scattered foreign body giant cells can be observed next to mononuclear cell infiltrates. In addition, in this group, a capsule formation can be observed around the implantation site. Worth mentioning is the fact that some vessel structures can be observed in the transition area (scaffold-to-tissue, ROI quadrant A, Figs 7 and 8) in the group DFO + no cells. On the other hand, in the group where the scaffold was seeded with cells that was exposed to 120 μM DFO, less infiltration of inflammatory cells was observed. Indeed, slightly less infiltration of granulocytes is seen in this group when compared to the rest of the studied groups. Furthermore, the cellular infiltration is scattered throughout the scaffold. This is in contrast to, for instance, the group with DFO + no cells, in which the infiltration decreased towards the center of the scaffold. In the environmental hypoxia group, multinucleated foreign body giant cells are present besides a dense infiltration of leukocytes. This infiltration is occupying approximately 70% of the scaffold material. Interestingly, the normoxic control group presented characteristics more similar to the 120 μM DFO group, with a scattered infiltration of granulocytes. Amorphous collagen struts can be seen that are thinner than in the other samples.

## Discussion

The seeding density of the collagen-GAG scaffold was determined by measuring the metabolic activity in regards to the cellular proliferation of AdMSCs over time. The concentration determined to be the most suitable in regards to quick and stable cellular growth without loss of activity overtime was 5 × 10^5^ AdMSCs per 10 mm collagen-GAG disc (Fig. 1), equivalent to 6.4 × 10^5^ cells per cm^2^. This information could give insight into the optimal cellular density for larger scaffolds for translation into clinical application.

After determining the optimal seeding density, the AdMSCs were exposed to either 1% O_2_ or a variety of concentrations of DFO. While there was minimal difference in expression after 12 hours between environmental hypoxia and cells exposed to 120 μM DFO, those exposed to 30 or 60 μM of DFO showed lower expression of HIF-1α indicating that it is concentration dependent. HIF-1α activity is suppressed in diabetics due to the high-glucose levels and, as a result, impairs wound healing^19^. Thangarajah *et al*. found that therapeutic doses of DFO could create HIF-1α activity and, therefore, a release of VEGF capable to improve angiogenesis^19^. Wang *et al*. showed that local injections of DFO enhanced healing in diabetic skin flaps in mice, similar to non-diabetic animals, through an increased production of HIF-1α and VEGF^20^. Hou *et al*. also found that by treating HUVECs with DFO in diabetic rats that there was an increased level of angiogenesis and wound healing^21^. Furthermore, Zhang *et al*. found that irradiated mice treated with DFO showed increased healing and function of salivary glands^22^. Elevated HIF-1α levels lead to an upregulation of VEGF, as well as several other genes, such as erythropoietin, increased cell survival, and glycolysis^23^,^24^, which would in turn lead to improved healing. In this study, we saw that cells exposed to 120 μM DFO showed similar levels of HIF-1α to those cultured under hypoxic oxygen tensions indicating that the chemical would make a suitable replacement for specialized incubators and chambers.

The release of VEGF triggers endothelial cells to migrate and proliferate in order to form new immature vessels^25^. *In vitro,* there was an increase in VEGF release with cells exposed to 120 μM DFO after three days when compared to the other cells which may indicate a higher release of HIF-1α at a later and untested time point. The addition of hypoxia and hypoxia-mimetic conditions enhanced levels of VEGF, which could also enhance MSC migration and, furthermore, VEGF has been shown to be the critical mediator for MSCs towards angiogenesis^26^.

*In vivo,* the release of VEGF did not significantly differ between cells treated with normoxic and hypoxic conditions (Fig. 6a). The cells under hypoxic conditions may have experienced stress during the implantation by suddenly being exposed to normoxic conditions. As the environmental hypoxic effects would soon be lost and the measurement was taken 14 days after implantation, a combination of cell stress, which may have led to cellular death, could lessen the effects of the pre-conditioning when compared to those from the cells exposed to normoxic conditioning. This would indicate that the cells were altered from the sudden change in oxygen tensions and mean cells conditioned under environmental hypoxia are an unpredictable treatment. As DFO is taken up by cells the outcome would not be effected upon implantation, which was seen in significantly increased VEGF concentrations *in vivo*.

In wound healing, SDF-1α is responsible for recruiting MSCs and their release of growth factors to the injured tissue, as well as, increasing wound healing rates and neovascularization^27^. The release of SDF-1α *in vitro* was significantly higher after seven days when the AdMSCs were treated with DFO (Fig. 3b) and was not concentration dependent. With environmental hypoxia, the release was lower than cells exposed to normoxic conditions. Although it has been found that BMMSCs lose CXCR4, the receptor for SDF-1α^28^ *in vitro,* Najafi *et al*. found treating the cells with DFO could increase the expression of CXCR4 *in vitro*^29^. It is not surprising then that there was such a significant increase of SDF-1α *in vitro* from the AdMSCs treated with DFO, as CXCR4 is the receptor for SDF-1α. Moreover, the release of SDF-1α was lower in cells treated with environmental hypoxia than those treated under normoxic conditions. Jing *et al*. also found a downregulation of SDF-1α with BMMSCs in hypoxic conditions^30^, suggesting that oxygen tensions similar to their niche *in vitro* are not useful to the release of chemotactic factors. On the other hand, there was no significant difference of SDF-1α expression between treatments *in vivo* (Fig. 6b). Liu *et al*. found that hypoxia preconditioned MSCs had upregulated the expression of SDF-1α when compared to those treated in normoxic conditions^31^. They also found, in subsequent treatments, that the differences in levels of SDF-1α peaked at 48 hours and became comparable after seven days^31^. As the data mentioned here was analyzed 14 days after implantation, further differences may be visible at earlier time points.

Without vascularization, transplanted cells will undergo apoptosis; therefore, the transplanted cells need to be close to the damaged vasculature of a wound in order to survive^32^. In the mouse model utilized, there was a visually higher density of neovascularization with hypoxic treatments when compared to normoxic treatments as analyzed after 14 days. Furthermore, there seems to be a higher instance of neovascularization with cells than with no cells at all. In the mouse model used in this research, the skin was removed and the scaffolds were placed over undamaged muscle tissue, which is highly vascularized, this could have facilitated AdMSC survival and growth factor release. For chronic wound treatment in a patient, necrotic tissue and debris need to be removed and the underlying tissue and muscle may provide enough vasculature for the transplanted AdMSCs to thrive. This analogy supports the use of this mouse model for chronic wound simulation.

Inflammation is an important stage in wound healing and needs to occur for successful healing. Chronic wounds remain in a state of prolonged inflammation that could be due to both local and systemic defects^33^. Interestingly, IL-12p70 was exhibited in small amounts from all treatments (Fig. 4). IL-12p70 stimulates T cells to produce IFN-γ, a cytokine activated by inflammation, which could reduce an occurrence of fibrosis^34^. Knockout IFN-γ and IL-10 mice exhibit increased epithelialization, angiogenesis, and collagen deposition resulting in increased wound closure^33^,^35^. Therefore, the lack of expression of these cytokines produce a desired effect, but while IFN-γ is necessary to viral infections, it can be toxic against other infections^36^.

IL-6 can be either a pro- or anti-inflammatory cytokine^37^ but is crucial in wound healing by way of impaired angiogenesis, leukocyte recruitment, and collagen deposition^38^. Furthermore, Lin *et al*. have demonstrated that IL-6 knockout mice exhibit delayed wound healing^38^. In addition, Gallucci *et al*. also found a decreased inflammatory response, reduced re-epithelialization, and reduced granulation tissue formation with these mice^39^. MCP-1 induces endothelial cell migration and VEGF-mediated angiogenesis^40^,^41^. MCP-1 knockout mice experience delayed re-epithelialization, angiogenesis, and collagen synthesis^42^. The significantly high expression of IL-6 and MCP-1 from mice receiving the DFO treated cells (Fig. 4) indicates that there is an increase in healing through inflammatory cytokine expression.

TNF expression has been shown to be present in situations that inhibit^33^ and enhance^43^,^44^ wound healing. Due to the expression of the other inflammatory cytokines in this work, it can be assumed that the TNF expression exhibited provides a favorable result. However, in elderly patients an increased level of IL-6 and TNF can be associated with a decrease of necessary wound healing growth factors^2^.

The histological observations confirmed some of the results obtained *in vitro* and *in vivo* in our study. Higher amounts of small capillaries were found in the animals exposed to 120 μM DFO when compared to the others studied. In addition, in this group, treated AdMSCs both, *in vitro* and *in vivo*, released a higher amount of VEGF. This clearly indicates the positive effect of DFO over environmentally induced hypoxia on vascularization of the implanted scaffolds. This seems to be mediated by the secretion of VEGF from the implanted AdMSCs. On the other hand, there are indeed some systemic signs of inflammation in the mice exposed to 120 μM DFO. This can be concluded as *in vivo* levels of IL-6 and MCP-1 are significantly higher in this group when compared to environmental hypoxia and normoxic control groups. However, histological sections show less infiltration of inflammatory cells when compared to all other studied groups, except for the normoxia group where it was similar. Clear signs of rejection, with capsule formation, were observed in the control group where the scaffold was implanted alone without AdMSCs and without further treatment.

In this work, a collagen-GAG scaffold was used to deliver preconditioned AdMSCs either in low oxygen tensions, as they exist in their niche, or HIF-1α stabilizing DFO in order to facilitate wound healing. The healing capabilities were assessed by the angiogenic potential and cytokine release via protein expression. The pattern of systemic inflammation related factors matched the ones previously described to increase wound healing, indicating DFO could be a promising alternative to environmentally initiated hypoxia making clinical translation easier. While these values were favorable for DFO treated cells, the local effects could also be examined to determine any possible differences. In addition, a more detailed analysis of the interaction of the scaffolds and cells in the host environment by way of additional growth factor analysis at additional time points *in vivo* would be beneficial.

DFO has been used in clinical settings and should demonstrate a reasonably applicable wound healing treatment for diabetics, the elderly, and immuno-impaired patients with the methods evaluated. With the right resources, the culture and expansion of AdMSCs with DFO may improve the angiogenic and wound healing potential by providing a more stable environment for the cells after isolation through the point of implantation. Although, in order to obtain a homogeneous culture, the isolation and maintenance of the cells still remains an issue that needs to be handled in a laboratory setting. Additional animal experiments with those that have impaired healing such as elderly or diabetic animals at longer time points and larger animals, such as pigs, would be beneficial to see the effects after full wound healing.

While chronic wounds are a clinical issue where the methods for healing require improvement, this study offers insights into the delivery of AdMSCs to injured tissue via a scaffold. Therefore, this data helps to develop new strategies for the treatment of other tissue defects that could benefit from cell-seeded scaffold healing, such as internal damages related to stroke, heart disease, or non-healing bone fractures.

## Materials and Methods

### Human adipose-derived stem cell isolation and culture

Lipoaspirates were donated from female patients undergoing fat removal procedures who had given informed consent to participate at the Department of Plastic Surgery and Hand Surgery in the University Hospital rechts der Isar (Munich, Germany). Furthermore, all experimental protocols were approved by the ethical committee of the University Hospital rechts der Isar and were carried out in accordance with the approved guidelines. Phosphate buffered saline (PBS; Biochrom, Berlin, Germany) containing 0.1% (w/v) collagenase A (Roche, Basel, Switzerland) was added to the lipoaspirates in equal volumes and incubated at 37 °C for 30 min. The collagenase digestion was stopped with Alpha Modified Eagle’s Medium with nucleosides and 2 g/L NaHCO_3_ (αMEM; Biochrom), centrifuged, and the supernatant was aspirated. The cell pellet was resuspended with PBS, filtered through a 100 mm cell strainer, further centrifuged, and the resulting stromal vascular fraction was plated in αMEM, supplemented with 10% (v/v) heat inactivated male AB human serum (Sigma-Aldrich, St. Louis, MO, USA) and 1% (v/v) antibiotic/antimycotic solution (PAA, Pasching, Austria) under standard cell culture conditions (37 °C, 5% CO_2_). All experiments were conducted with cells from passage three in triplicate (n = 3) and from three donors (N = 3).

### Cell seeding and concentration

To determine the optimal seeding density for 10 mm (0.22 mm thick with a fluid capacity of 143 ± 5.6 μl/cm^2^ 13) collagen-GAG scaffolds (Integra LifeScience Corp.; Plainsboro, NJ, USA) comprised of a 92:8 ration of type I bovine tendon collagen:chondroitin 6-sulfate were used. AdMSCs were seeded dropwise in concentrations of 5 × 10^3^, 5 × 10^4^, 5 × 10^5^, and 1 × 10^6^, in 50 μL of supplemented αMEM, for comparison. The cells were allowed to adhere for 3 hours in standard cell culture conditions. After which, 1 ml of supplemented αMEM was added.

The metabolic activity of the AdMSCs on the scaffolds was examined using an alamarBlue fluorometric assay (AbD Serotec, Raleigh, NC, USA), which was incubated with the cells for 4 hours before analysis at 1, 7, and 14 days following manufacturer’s instructions. Scaffolds were then rinsed with sterile PBS and collected in 1x passive lysis buffer (PLB; Promega, Madison, WI, USA), chilled for 10 min at 4 °C, and pulse sonicated (SONICS Vibracell VCX130 Ultrasonic Cell Disrupter, Sonics & Materials, Inc., Newton, CT, USA) 10 times at 40% power to lyse the cells. To determine the amount of cellular proliferation, double stranded DNA was quantified using a PicoGreen assay kit (Invitrogen, Carlsbad, CA, USA) with the cell lysates from the same scaffolds.

### Hypoxia induction and DFO exposure

After cells were seeded onto the collagen-GAG discs and allowed to adhere for 3 hours, they were placed into a hypoxic incubator with 1% O_2_ or incubated under standard cell culture conditions with deferoxamine mesylate (DFO; Sigma-Aldrich) added to final concentrations of 30, 60, or 120 μM. Scaffolds seeded with AdMSCs cultured under standard conditions were used as a control.

### HIF-1α quantification

To determine the HIF-1α concentrations, scaffolds were collected after 12 and 24 hours, rinsed twice with sterile PBS, snap frozen with liquid nitrogen, and stored at −80 °C until analysis. The total HIF-1α concentration was evaluated using an ELISA kit (Human/Mouse Total HIF-1α; R&D Systems, Minneapolis, MN, USA) according to manufacturer’s instructions. Total protein was quantified with a BCA assay (Thermo Scientific, Waltham, MA, USA).

### VEGF and SDF-1α release *in vitro*

To determine the release of VEGF and SDF-1α, the medium was collected from the scaffolds after 1, 3, and 7 days, snap frozen with liquid nitrogen, and stored at −80 °C until analysis. The total concentrations released were determined using a Human VEGF Quantikine ELISA kit and Human CXCL12/SDF-1 alpha Quantikine ELISA kit (R&D Systems), respectively, according to manufacturer’s instructions. Due to the high sensitivity of the assay, only 5 μL of the conditioned medium was used in the VEGF analysis.

### Mouse wound healing model

The local legislative committee of the state of Bavaria (Regierung von Oberbayern) and approved all experimental procedures. Additionally, all used procedures were in accordance with the Guide for the Care and Use of Laboratory Animals as defined by the National Institute of Health and the German animal welfare act (TierSchG).

Male hairless immunocompetent SKH1-Elite mice (Charles River, Sulzfeld, Germany) were used with four mice per group (N = 4). Mice were allowed to adjust to the facility for one week before the operation. Each scaffold treatment was maintained in their respective cell culture conditions for 24 hours before surgery. The groups consisted of scaffolds incubated in only medium or medium with 120 μM DFO and those with AdMSCs incubated in normoxic conditions, environmentally induced hypoxic conditions (1% O_2_), or with 120 μM DFO (Table 1).

Animals were fully anesthetized by inhalation of isofluorane (CP Pharmaceuticals Ltd., Wrexham, UK) and 0.07 ml of buprenorphine was administered subcutaneously before wounding. Animals were kept warm with a heat pad and their eyes were dabbed with a lubricating ointment for the duration of the surgery. Wounding was done with an 8 mm skin biopsy punch (Acuderm, Fort Lauderdale, FL, USA) on the left and right side of the back. A titanium mesh (TiMesh extralight tetanized mesh; Nürnberg, Germany) was placed in the wound to avoid wound contracture and the scaffold was sutured (Ethilon nylon monofilament sutures 5–0; Ethicon, Norderstedt, Germany) into place. A vacuum assisted closure (V.A.C.) Therapy Dressing (KCI Medical Products, Wimborne Dorset, UK) was then sutured in place over the wounded areas. Medical tape (Hansaplast, Hamburg, Germany) was further wrapped around the torso of the mouse to protect the wounded area and 0.03 ml of diazepam was administered subcutaneously before allowing the animal to recover near a heat lamp. Animals were checked daily for well being and given a 0.07 ml injection of buprenorphine as well as 0.03 ml of diazepam every three days.

After 14 days, the animals were euthanized with an overdose of isofluorane. The chest cavity was opened and blood was extracted from the heart which was then were placed on ice for 30 min. Blood samples were then centrifuged at 5,000 × g for 10 min. The serum was moved to a new tube and stored at −80 °C until analysis. All bandages and sutures were removed and the animal was imaged. Then the skin of the back was cut away and the scaffolds were imaged with a transilluminator connected to a stereoscope optical microscope (Zeiss, Jena, Germany). The scaffolds were cut from the skin and half was stored in 3.7% formaldehyde for sectioning while the other half was snap frozen in liquid nitrogen and stored at −80 °C until further analysis.

### Digital segmentation

The vascular density of the harvested scaffolds was segmented from images obtained by transillumination using Vessel Segmentation and Analysis (VesSeg) software v0.1.4 (http://www.isip.uni-luebeck.de/index.php?id=150)^45^. Images were converted to gray scale and via a semi-automated process, a threshold was set to enhance vessel visualization. The images were further segmented so every white pixel was assigned to vessel-like structures and the rest was assigned as background in black. Further caution was taken in order to eliminate false-positive and false-negative structures.

### Histological assessment

The scaffolds were fixed with 3.7% formaldehyde for at least 24 hours. Subsequently, the scaffolds were dehydrated using gradually increasing concentrations of ethanol and embedded in paraffin. Sections (5 μm) of each scaffold were obtained and then stained with hematoxylin and eosin (H&E) and Masson’s Trichrome following standard protocols. The slides were observed and photographed with a microscope (Biorevo BZ9000, Keyence, Osaka, Japan) at different magnifications. A general picture of the entire histological section was performed using the software BZ-II Viewer and BZ-II Analyzer (Keyence).

### Inflammatory protein levels

Serum collected from the mouse heart after 14 days was used to determine if there was a systemic inflammatory response to the treatments. A BD Cytometric Bead Array (CBA) Mouse Inflammation Kit (BD Biosciences; San Jose, CA, USA) was utilized in order to simultaneously measure levels of Interleukin-6 (IL-6), Interleukin-10 (IL-10), Monocyte Chemoattractant Protein-1 (MCP-1), Interferon-γ (IFN-γ), Tumor Necrosis Factor (TNF), and Interleukin-12p70 (IL-12p70) with a BD FACSCanto™ II flow cytometer (BD Biosciences). Data was analyzed using FCAP Array™ Software v3.0 (BD Biosciences).

### VEGF and SDF-1α release *in vivo*

To determine if the AdMSCs were still present and releasing growth factors, human VEGF and SDF-1α protein concentrations were measured from scaffolds collected from mice 14 days after surgery. The cells in the scaffolds were lysed with 300 μL lysis buffer (R&D Systems recipe for Lysis Buffer #11:50 mM Tris (pH 7.4), 300 mM NaCl, 10% (w/v) glycerol, 3 mM EDTA, 1 mM MgCl_2_, 20 mM β-glycerophospate, 25 mM NaF, 1% Triton X-100, 25 μg/ml Leupeptin, 25 μg/ml Pepstatin, and 3 μg/ml Aprotinin), pressed with a pestle, vortexed shortly, and left for 10 min on ice. Then the scaffolds were pressed once more with a pestle, shortly vortexed, and centrifuged for 5 min at 3000 × g at 4 °C. The resulting supernatant was analyzed with VEGF and SDF-1α ELISAs (R&D Systems) where the VEGF was diluted 1:20, as with the *in vitro* analysis.

### Statistical analysis

Results were analyzed with GraphPad Prism^®^ version 6.0e for Mac OSX (GraphPad Software, San Diego, CA USA) and are shown as mean ± standard deviation. Significant differences between sample groups were determined by analysis of variance (ANOVA) with a Bonferroni post-test where *p* ≤ 0.05 was considered statistically significant. Asterisk denoting statistical significance are signified by: *p < 0.05, **p < 0.01, ***p < 0.001, and ****p < 0.0001.

## Additional Information

**How to cite this article**: Wahl, E. A. *et al*. VEGF released by deferoxamine preconditioned mesenchymal stem cells seeded on collagen-GAG substrates enhances neovascularization. *Sci. Rep.* **6**, 36879; doi: 10.1038/srep36879 (2016).

**Publisher's note:** Springer Nature remains neutral with regard to jurisdictional claims in published maps and institutional affiliations.

## Figures and Tables

**Table 1:** Experimental groups and animal allocation.

*In vitro*		*In vivo*	
Group	N (donor)/n (assay replicate)	Group	N (animal per group) with 2 scaffolds in each
AdMSCs/Collagen-GAG scaffold/**1% O**_**2**_ (environmental hypoxia)	3/3	Collagen-GAG scaffold (**no cell** control)	4
AdMSCs/Collagen-GAG scaffold/**30 μM DFO** (normoxia)	3/3	Collagen-GAG scaffold + DFO (**DFO + no cells**)	4
AdMSCs/Collagen-GAG scaffold/**60 μM DFO** (normoxia)	3/3	AdMSCs/Collagen-GAG scaffold/**120 μM DFO** (normoxia)	4
AdMSCs/Collagen-GAG scaffold/**120 μM DFO** (normoxia)	3/3	AdMSCs/Collagen-GAG scaffold/**1% O**_**2**_ (environmental hypoxia)	4
AdMSCs/Collagen-GAG scaffold/**21% O**_**2**_ (normoxia)	3/3	AdMSCs/Collagen-GAG scaffold/**21% O**_**2**_ (normoxia)	4
